# Associations among Stress, Anxiety, Depression, and Emotional Intelligence among Veterinary Medicine Students

**DOI:** 10.3390/ijerph18083934

**Published:** 2021-04-08

**Authors:** Julia Wells, Kylie Watson, Robert E. Davis, Syed Siraj A. Quadri, Joshua R. Mann, Ashutosh Verma, Manoj Sharma, Vinayak K. Nahar

**Affiliations:** 1 Center for Animal and Human Health in Appalachia, College of Veterinary Medicine, Lincoln Memorial University, Harrogate, TN 37752, USA; julia.wells@lmunet.edu (J.W.); kylie.watson@lmunet.edu (K.W.); ashutosh.verma@lmunet.edu (A.V.); 2 Substance Use and Mental Health Laboratory, Department of Health, Human Performance and Recreation, University of Arkansas, Fayetteville, AR 72704, USA; red007@uark.edu; 3 DeBusk College of Osteopathic Medicine, Lincoln Memorial University, Knoxville, TN 37932, USA; syed.quadri@lmunet.edu; 4 Department of Preventive Medicine, School of Medicine/John D. Bower School of Population Health, University of Mississippi Medical Center, Jackson, MS 39216, USA; jmann4@umc.edu; 5 Department of Environmental & Occupational Health, School of Public Health, University of Nevada, Las Vegas, NV 89119, USA; manoj.sharma@unlv.edu; 6 Department of Dermatology, School of Medicine, University of Mississippi Medical Center, Jackson, MS 39216, USA

**Keywords:** veterinary students, mental health, anxiety, depression, stress, emotional intelligence

## Abstract

**Background:** Veterinary students are faced with immense pressures and rigors during school. These pressures have contributed to elevated levels of stress, anxiety, and depression (SAD) among veterinary students relative to the general population. One proposed concept to help students combat SAD is that of emotional intelligence (EI). We explored the relationship between EI and SAD among veterinary students at a college in the Southeast United States. **Methods:** A cross-sectional study design was implemented among a convenience sample of 182 veterinary medical students. The survey instrument contained 56 items that elicited information about students’ demographics, perceived stress, anxiety, and depression, and emotional intelligence levels. Data analysis included univariate statistics, Pearson’s correlations, and multiple regression and independent samples *t*-tests. **Results:** The study revealed a statistically significant, negative correlation between EI levels and stress, anxiety, and depression. Additionally, a statistically significant, positive correlation was found between stress and anxiety as well as both stress and anxiety and depression. Multiple linear regression showed that EI was a statistically significant predictor of stress (*b* = −0.239, *p <* 0.001), anxiety (*b* = −0.044, *p* < 0.001), and depression (*b* = −0.063, *p* < 0.001), after controlling for sociodemographic variables. Students’ *t*-test results revealed a statistically significant mean difference in EI scores among students screening positive versus negative for depression, with students screening negative having a mean EI score of 10.81 points higher than students who screened positive for depression. **Conclusion:** There is a scientifically supported need for interventions in veterinary school to integrate EI into the veterinary medical curriculum and consider the EI levels of veterinary student candidates.

## 1. Background

Veterinary school is an intensive and rigorous program. Due to these rigors, concerns have risen for the mental well-being of veterinary students, specifically evaluation of the prevalence, impact, and management of stress, anxiety, and depression (SAD) among students [1]. Data has shown a significant increase in SAD amongst veterinary students compared to the general population [1]. In one study, researchers concluded that academic stress was one of the main stressors impacting veterinary medical students’ well-being and it was associated with deleterious outcomes such as depression and anxiety [2]. In a survey conducted in 2019 by Nahar et al., 22.6% of veterinary students screened positive for depression and 52.3% for generalized anxiety [1]. These findings are similar to those of another study that concluded one-third of first-year veterinary students reported depression levels above the clinical cut-off [3] and another that concluded 49–69% of veterinary students reported at or above clinical depression levels [2]. As significant correlations exist between stress, anxiety, and depression [4], such interrelationships suggest that veterinary students increasingly experience significant levels of SAD. When comparing the levels of psychological distress of veterinary students and medical students, among other groups, veterinary students experienced higher levels of stress [5].

The concerns of SAD in veterinary students do not end at graduation but expand into their professional lives as practicing veterinarians [1]. In 2012, 701 licensed veterinarians in Alabama participated in a study that concluded that 66% of respondents were clinically depressed and 24% of veterinarians stated they had contemplated suicide since beginning veterinary school [6]. Another study concluded that 9% of veterinarians had serious psychological distress, 31% had experienced depressive episodes since leaving veterinary school, and 17% experienced suicidal thoughts [7]. With these results in mind, it is important that interventions to address and manage SAD within the veterinary profession be implemented in veterinary school.

Emotional intelligence (EI) is a specific form of social intelligence that involves the capability to monitor and regulate personal feelings along with the feelings of others [8]. In addition to monitoring and regulating feelings, EI includes the ability to differentiate these feelings and assess one’s own thinking and behavior accordingly [8]. While EI may initially appear unrelated to psychological conditions such as SAD, a previous study concluded that EI is an important factor in mediating the relationship between unfortunate events and states of psychological distress [9]. Researchers found a negative correlation between EI and psychological distress among nursing students, suggesting that higher stress levels indicated lower EI [9]. Research on the importance of EI in human medical schools has discovered that higher EI is associated with both lowered stress levels [10] and improved academic performance [11] in medical students. Limited research has been performed on EI in veterinary medical students. In a cross-sectional study of fourth-year veterinary students, interns, and residents, researchers found consistently lower EI levels in these participants compared to other professionals [12]. Additionally, the EI scores across the various groups were similar and did not increase with training level [12]. This study is valuable in that it provides baseline EI data among different veterinary populations in the five composite areas of EI: self-perception, self-expression, interpersonal skills, decision-making, and stress tolerance [12]. Additional research has suggested that, although implementation may be difficult, EI is a valuable concept that should be incorporated into veterinary education to improve student communication skills among other “non-technical competencies” [13]. Professionally, veterinarians with higher EI could elicit improved client satisfaction and client compliance with treatment plans [13]. With these potential benefits in mind, the author stresses the importance of completing further research studies on the implementation and benefits of EI in veterinary students [13]. Changing the veterinary curriculum to incorporate EI training without research studies supporting the importance of EI could waste valuable resources and cause undue stress to students [13].

While numerous researchers have investigated the prevalence and effects of stress, anxiety, and depression on veterinary students and their education [1,3,14], limited data exist on the impact of EI on veterinary student education [12,13]. EI has been studied to a further extent in other healthcare professions [10,11]; however, studies indicate veterinary students, specifically, have significantly higher levels of stress compared to medical students [5]. Therefore, the results of studies on medical students should not be extrapolated as applicable to veterinary students specifically. Furthermore, the veterinary professionals have long combatted serious problems with depression, suicide, and psychological distress [1,6,7]. When considering all of these factors together, it is clear that a study focusing exclusively on the correlations among stress, anxiety, and depression on EI within the veterinary student population is warranted.

Within the aforementioned context, continued study of EI among veterinary students may immensely aid in the development of strategies aimed at mitigating deleterious psychological and behavioral concerns associated with the management of stressors encountered during veterinary school. Thus, the purpose of this study was to examine the relationships between EI and SAD in veterinary students at a veterinary college in the southeast region of the United States.

## 2. Methods

### 2.1. Study Design, Sampling, and Procedure

Researchers applied a cross-sectional design to survey a convenience sample of veterinary students at a college in the southeast United States. Students were recruited via email to complete a 56-item web-based questionnaire. Utilizing the questionnaire, researchers assessed students’ sociodemographic attributes in addition to stress, anxiety, depression, and EI. Participation in the study was voluntary, and students were informed that they could withdraw from the study at any time. Data collection took place over 3 weeks in March 2020, with reminder emails sent one and two weeks following initial recruitment. The study was granted full ethics approval by the university’s Institutional Review Board (IRB; Protocol # 868 V.0).

### 2.2. Survey Instrument

The survey instrument utilized consisted of 56 items. The first 9 items assessed the socio-demographic data of the students, specifically gender, age, ethnicity, student status, grade point average, employment, and housing. The remainder of the questionnaire consisted of three separate scales: a 10-item perceived stress scale (PSS) [15], a 4-item patient health questionnaire (PHQ-4) to assess anxiety and depression [16,17], and a 33-item scale to assess EI [18]. The PSS consisted of questions such as “In the last month, how often have you felt nervous and ‘stressed’?”. Participants responded on a 5-point scale (0 = Never to 4 = Very often). The PHQ-4 required students to respond with how often in the past two weeks they have felt bothered by problems such as “feeling nervous, anxious, or on edge”. Students responded on a 4-point scale (0 = Not at all to 3 = Nearly every day). The 33-item EI scale consisted of statements such as “I know when to speak about my personal problems to others” and “When my mood changes, I see new possibilities”. Students scored their agreement with the statements using a 5-point scale (1 = strongly disagree to 5 = strongly agree). Student responses to all items were anonymous, and the survey instrument was designed to take 10–15 min to complete.

The PSS, which has been validated, is the most commonly used psychological tool for assessing stress perception [15]. In the present study, the value of Cronbach’s alpha (i.e., internal consistency reliability) for PSS was 0.839. The PHQ-4 is an ultra-brief and valid instrument for measuring both anxiety and depressive disorders [16]. In the present study, the value of Cronbach’s alpha for PHQ-4 was 0.855. The 33-item EI scale [18] was developed by Schutte et al. (1998) based on a cohesive and comprehensive model of emotional intelligence by Salovey and Mayer (1990) [18]. The 33-item EI scale, according to Schutte et al. (1998) [18], holds promise as a reliable and valid measure of emotional intelligence, as conceptualized by Salovey and Mayer (1990) [8]. The Cronbach’s alpha coefficient for EI scale was 0.875 in the present study.

### 2.3. Data Analysis

All data analyses were performed using the Statistical Package for Social Science (SPSS) v. 25. Researchers first calculated univariate statistics to describe student characteristics and variables of interest. Next, Pearson’s correlations were determined to examine the relationship, or lack thereof, between EI and the psychological variables of interest. Three multiple linear regression models were conducted to determine the association between EI and stress, anxiety, and depression, after controlling for sociodemographic variables. Only those variables that showed a statistically significant bivariate association with stress, anxiety, and depression were controlled in the multiple regressions. Post hoc power analyses with a medium effect size of 0.15 and alpha level of 0.05 indicated an achieve power of 0.99, 0.99, and 0.98 for the stress model, anxiety model, and depression model, respectively. The relationship between EI and depression and anxiety was further explored through the creation of dichotomous variables indicating the presence of a potential depressive or anxiety disorder using established PHQ-4 cut points [16]. Independent samples *t*-tests determined the presence or absence of a statistically significant difference in EI scores between those who screened positively and negatively for depression and anxiety disorders. Statistical significance was set at an alpha level of 0.05, determined *a-priori*.

## 3. Results

A total of 182 students of veterinary medicine participated in the study. Table 1 displays the participants’ demographic breakdown. The mean age of participants was 25.2 years old, and the majority identified as female (85.7%). Most participants self-identified as Caucasian (89.0%), and the breakdown among first-, second-, third-, and fourth-year students was 34.1%, 25.3%, 26.4%, and 11.0%, respectively. The majority of respondents lived off campus (93.4%), and most were unemployed (77.5%). Finally, the most frequently reported grade point averages were 3.00–3.49 (39.0%), followed by 3.50–4.00 (34.6%) and 2.50–2.99 (15.9%).

Table 2 presents the zero-order correlations among the four psychological variables studied: EI, stress, anxiety, and depression. Statistically significant, negative correlations were found between EI and stress (*r* = −0.417), EI and anxiety (*r* = −0.299), and EI and depression (*r* = −0.450). There were statistically significant, positive correlations between stress and anxiety (*r* = 0.734), stress and depression (*r* = 0.636), and anxiety and depression (*r* = 0.590). All correlations were said to be significant at a *p*-value of less than 0.001. As shown in Table 3, EI was a statistically significant predictor of stress (*b* = −0.239, *p* < 0.001), anxiety (*b* = −0.044, *p* < 0.001), and depression (*b* = −0.063, *p <* 0.001), after controlling for sociodemographic variables.

Table 4 displays the results of *t*-tests performed to assess the differences in EI among respondents who screened either positive or negative for possible anxiety/depressive disorders. More respondents screened positive for anxiety than negative (*n* = 113 Anxiety, *n* = 45 No anxiety), while more screened negative for depression than positive (*n* = 97 No depression, *n* = 61 Depression). Regarding anxiety, a mean difference of 5.29 was found between the EI scores of participants screening positive (M = 115.31, SD = 13.03) and negative (M = 120.60, SD = 13.09). For depression, a statistically significant, mean difference of 10.81 was calculated between participants screening positive (M = 110.18, SD = 11.21) or negative (M = 120.99, SD = 12.72) for depression.

## 4. Discussion

While there has been much research on stress, anxiety, and depression among veterinary students, very little has been done to assess their emotional intelligence and the role it plays in managing SAD. The objective of this study was to examine the relationships between SAD and EI in veterinary students. Data were collected via a 56-item web-based questionnaire that was sent to students at a veterinary college in the southeast region of the United States. Using the results of this study, researchers hope to develop and refine stress management interventions in colleges of veterinary medicine by incorporating EI into such interventions. Future studies could investigate what other variables of the academic and family context of the students intervene for a more complete and effective intervention, and avoid as much as possible selective processes that leave out some students.

A major finding in this study revealed a statistically significant, negative correlation between EI levels and stress, anxiety, and depression levels among participants. This relationship indicates that veterinary students with higher levels of EI experience lower levels of SAD, whereas students with lower levels of EI tend to exhibit higher levels of SAD. These findings mirror those of another study that found higher EI to be associated with better academic performance as well as lower self-perceived stress among medical undergraduates [19]. While the findings align, these studies differ in target population given that our study focused exclusively on graduate-level veterinary students and the latter studied undergraduate-level medical students [19]. Additionally, our findings were in contrast with those of one study that found that during surgical procedures, medical students with higher EI were more likely to experience stress during an unfamiliar surgical procedure [20]. A possible explanation for the difference in findings lies in the unique circumstances under which data were collected in each study. While we assessed general well-being and overall SAD via a survey, Arora et al. studied medical students’ stress before, during, and after the completion of a surgical task via self-reporting and heart rate monitoring [20].

Another major finding in this study revealed a statistically significant mean difference in EI scores of veterinary students who screened for depression. Those who screened positive for a potential depressive disorder had a mean EI score of 10.81 points lower than students who screened negative. Such findings propose a link between EI and depression, suggesting that higher EI would likely result in decreased depression, while lower EI correlates with an increased incidence of depression. Our findings are similar to those in a study that found that clinically depressed adults scored lower than non-depressed participants in various dimensions of EI [21]. Additionally, results from our research also mirror another study conducted on undergraduates in the Middle East that found a negative correlation between EI and depressive symptoms [22]. These additional studies in conjunction with our own results provide further evidence of a link between EI and depression.

The major findings of the study—a negative correlation among EI and SAD along with a mean difference in EI among students screening for depression—suggest a strong need for the consideration of EI in modern-day veterinary medical programs. The correlations supported by this study provide strong evidence to suggest that increasing EI in veterinary students will result in a decrease in SAD. Importantly, literature suggests that it is possible to increase one’s EI through intervention [23,24,25]. With this evidence in mind, we support the prior recommendation [13] to incorporate EI into veterinary school curriculum. One option to incorporate EI into the veterinary medical curriculum is to have professors teach EI during school as a clinical skill. For this to be successful, schools must seek faculty members with high EI themselves and potentially hire a specialist with a background in EI. Another manner by which EI may be integrated is to instill screening programs during the veterinary school interview process to seek students with elevated levels of EI relative to their peers. This will ensure veterinary schools are admitting emotionally intelligent students with the competency to handle the rigors of the curriculum. A final option, and perhaps the most realistic, is a blending of the two prior possibilities. If schools both screen for EI during interviews and integrate teaching EI to veterinary students into their curriculum, we believe veterinary students can become far better equipped to manage stress, anxiety, and depression while in school. 

In discussing the current study, there are several limitations to consider when evaluating the results. To begin, the researchers used a convenience sample of 182 veterinary students from a single veterinary school in the southeast United States. Therefore, the findings should not be extrapolated as representative of all veterinary students. Along those same lines, the demographic findings revealed that the majority of respondents were female and white/Caucasian. Therefore, findings should not be generalized as representative of all demographics. Aside from sampling and demographics, the general nature of self-reported data lends the study to bias and potential reporting inaccuracies due to forgetfulness or a willingness to report expected results. Finally, this was a cross-sectional study, meaning it provides merely a snapshot in time of veterinary students’ perceived levels of stress, anxiety, depression, and EI. Additional studies, likely in a more longitudinal format, would be necessary to generalize and extrapolate results in the future.

## 5. Conclusions

Our results revealed a statistically significant, negative correlation between EI levels and stress, anxiety, and depression in veterinary students. Additionally, we found a statistically significant, mean difference in EI of 10.81 among veterinary students screening negative versus positive for potential depressive disorders. These results support the necessity for EI to be strongly considered in veterinary medical curriculum. As a way to incorporate EI into veterinary school, we have recommended both screenings for EI during veterinary school interviews and teaching EI as a skill in the veterinary school curriculum. The ability to process and control stress, anxiety, and depression is critical to a student’s success in veterinary school, and our findings back up the idea that EI is a valuable tool to have in combating these mental health issues.

## Figures and Tables

**Table 1:** Descriptive Statistics (*n* = 182).

	M (SD)	*n* (%)
Age	25.2 (2.98)	
Gender		
Female		156 (85.7)
Male		20 (11.0)
Race/Ethnicity		
White/Caucasian		162 (89.0)
Non-White		13 (7.1)
University Status		
1st year veterinary student		62 (34.1)
2nd year veterinary student		46 (25.3)
3rd year veterinary student		48 (26.4)
4th year veterinary student		20 (11.0)
Living status		
On Campus		6 (3.3)
Off campus		170 (93.4)
Employment status		
Employed		35 (19.2)
Unemployed		141 (77.5)
Grade point average		
Less than 1.99		1 (0.5)
2.00–2.49		9 (4.9)
2.50–2.99		29 (15.9)
3.00–3.49		71 (39.0)
3.50–4.00		63 (34.6)

Percentages reported may not equal 100% due to missing data.

**Table 2:** Zero-order correlation matrix of psychological variables.

Measure	1	2	3	4
1. Emotional Intelligence	-	−0.417 *	−0.299 *	−0.450 *
2. Stress			0.734 *	0.636 *
3. Anxiety			-	0.590 *
4. Depression				-
Mean	116.82	23.07	3.65	2.34
SD	13.22	6.99	1.82	1.86

* All correlations are significant at *p* < 0.001.

**Table 3:** Multiple regressions of psychological variables onto emotional intelligence and demographic control variables.

	Stress				Anxiety				Depression			
	*b*	*SE*	*β*	*p*	*b*	*SE*	*β*	*p*	*b*	*SE*	*β*	*p*
EI	−0.239	0.038	−0.442	<0.001	−0.044	0.011	−0.317	<0.001	−0.063	0.010	−0.448	<0.001
Model	*R*^2^ = 0.249	*F* = 25.689		*p* < 0.001	*R*^2^ = 0.126	*F* = 11.160		*p* < 0.001	*R*^2^ = 0.247	*F* = 16.358		*p* = 0.001

In each model, demographic variables exhibiting significant bivariate relationships with dependent variables are controlled for. Stress model covariates: gender. Anxiety model covariates: gender. Depression model covariates: age and grade point average.

**Table 4:** Differences in emotional intelligence between those exhibiting positive and negative screens for the presence of anxiety and depression.

	*n*	Mean	SD	Mean Difference	*t*	*p*	Effect Size
Anxiety	113	115.31	13.03	5.29	2.30	0.023	0.405
No Anxiety	45	120.60	13.09				
Depression	61	110.18	11.21	10.81	5.44	<0.001	0.902
No Depression	97	120.99	12.72				

Groupings are based on positive and negative screening per Patient Health Questionnaire 4 (PHQ-4) cut points. *Note:* effect sizes measured as Cohen’s *d.*

## Data Availability

The datasets used and/or analyzed during the current study are available from the corresponding author on reasonable request.

## Abbreviations

SAD	Stress, Anxiety, and Depression
EI	Emotional Intelligence
IRB	Institutional Review Board
PHQ-4	Patient Health Questionnaire
SPSS	Statistical Package for Social Science
